# On the interrelation of multiplication and division in secondary school children

**DOI:** 10.3389/fpsyg.2013.00740

**Published:** 2013-10-10

**Authors:** Stefan Huber, Ursula Fischer, Korbinian Moeller, Hans-Christoph Nuerk

**Affiliations:** ^1^Knowledge Media Research CenterTuebingen, Germany; ^2^Department of Psychology, Eberhard Karls UniversityTuebingen, Germany

**Keywords:** simple multiplication, complex multiplication, simple division, complex division, arithmetic, skill level, development

## Abstract

Multiplication and division are conceptually inversely related: Each division problem can be transformed into as a multiplication problem and vice versa. Recent research has indicated strong developmental parallels between multiplication and division in primary school children. In this study, we were interested in (i) whether these developmental parallels persist into secondary school, (ii) whether similar developmental parallels can be observed for simple and complex problems, (iii) whether skill level modulates this relationship, and (iv) whether the correlations are specific and not driven by general cognitive or arithmetic abilities. Therefore, we assessed performance of 5th and 6th graders attending two secondary school types of the German educational system in simple and complex multiplication as well as division while controlling for non-verbal intelligence, short-term memory, and other arithmetic abilities. Accordingly, we collected data from students differing in skills levels due to either age (5th < 6th grade) or school type (general < intermediate secondary school). We observed moderate to strong bivariate and partial correlations between multiplication and division with correlations being higher for simple tasks but nevertheless reliable for complex tasks. Moreover, the association between simple multiplication and division depended on students' skill levels as reflected by school types, but not by age. Partial correlations were higher for intermediate than for general secondary school children. In sum, these findings emphasize the importance of the inverse relationship between multiplication and division which persists into later developmental stages. However, evidence for skill-related differences in the relationship between multiplication and division was restricted to the differences for school types.

## Introduction

Basic arithmetic operations constitute a milestone of numerical development. Children are introduced to addition, subtraction, multiplication, and division in elementary school and consolidate their arithmetic skills throughout secondary school. With schooling, they become more proficient and use more efficient strategies to solve both simple and complex arithmetic operations.

The focus of the current study is on multiplication and division, two of the four basic operations that are mathematically inversely related. By inversion of the operands, each division problem (e.g., 28 ÷ 4 = 7) can be recast as a multiplication problem (e.g., 4 × 7 = 28) and vice versa. In this study, we aimed to investigate how these two operations interact in fifth and sixth graders. Moreover, we specifically looked at how this interaction is influenced by skill level as the correlation between multiplication and division may not be the same for all children.

To establish how we expect these operations to interact, we will first give a brief overview of the most common strategies used to solve multiplication and division problems before discussing the role of skill level. While different strategies can lead to the correct result, these strategies are not equally efficient and also depend on the complexity of the problem. We will therefore start by outlining strategies used in simple operations before moving onto complex problems. Thereby, we will also pinpoint how the operations are interrelated.

Different types of multiplication problems are solved by applying different types of strategies. Simple multiplication problems (i.e., problems with single digit factors) are most often solved via fact retrieval from long term memory (e.g., Dehaene et al., 2003). Lemaire and Siegler (1995) reported that as early as grade 2, students' most common strategy to solve simple multiplication problems was the retrieval of multiplication facts. However, there is also evidence suggesting that not all multiplication problems are solved equally well. Multiplication problems with operands 0 or 1 are mostly solved by applying rules (Cooney et al., 1988). Other common findings for multiplications are the problem size effect, the tie effect and the five effect. The problem size effect describes the fact that reaction times and error rates for multiplication problems increase with increasing operands (Campbell and Graham, 1985). The tie effect reflects the finding that problems with equal operands (e.g., 4 × 4) are solved faster and with fewer errors than other problems (e.g., Campbell and Graham, 1985; Campbell and Gunter, 2002) Similarly, problems including 5 as one of the operands (e.g., 3 × 5) are solved faster and with fewer errors compared to other problems (five effect; e.g., Siegler, 1988).

When solving complex multiplication problems such as “7 × 16,” several steps of cognitive processing may be necessary (Geary and Widaman, 1987; Hope and Sherrill, 1987; Seitz and Schumann-Hengsteler, 2000): (1) Breaking down the multiplication problem (“7 × 10 = ?”, “7 × 6 = ?”); (2) retrieval of first fact (“7 × 10 = 70”) and storage of first partial result; (3) retrieval of second fact (“7 × 6 = 42”) and storage of second partial result; (4) addition of partial results (“70 + 42 = 112”). Thus, besides working memory capacities (for an overview, see Raghubar et al., 2010) and the ability to add the partial results, complex multiplication problems are solved by resorting to the retrieval of simple multiplication facts.

In contrast to multiplication, retrieval of division facts is not a very common strategy until grade 7. Robinson et al. (2006) explored Canadian children's usage of division strategies from grade 4–7 and found that from grade 4 onward, the reported frequency of the retrieval strategy stayed fixed at about 16%. The most common strategy of fourth graders was the addition strategy (i.e., adding up the divisor until the dividend is reached and counting how many times it was added). For instance, when solving “28 ÷ 4,” 4 would be added up 7 times (i.e., 4 + 4 + 4 + 4 + 4 + 4 + 4). However, from grade 5 onward the most frequently reported strategy was the division by mediation strategy. Application of this strategy involves relying on the inverse relationship between multiplication and division to solve simple division problems (e.g., “63 ÷ 7 = ?”→“7 × ? = 63”). The frequency of this strategy increases from 48.8% in grade 5–71.0% in grade 7.

In comparison to research on complex multiplication, research on complex division is even sparser (for an exception, see Hickendorff et al., 2010). Complex division problems usually refer to problems containing either a two-digit divisor (e.g., “90 ÷ 18 = 5”) or a two-digit quotient (e.g., “108 ÷ 6 = 14”). One way to solve complex division problems with a two-digit quotient is to split them up into multiple simple division problems, also known as the long division algorithm which was first described by Euclid (e.g., Heath, 1956). For instance, a typical strategy to solve “108 ÷ 6” would be: (1) Calculating how often 6 fits in 10 (once); (2) multiplying the result with the divisor (i.e., 1 × 6 = 6); (3) subtracting the result of step 2 from 10 (i.e., 10 − 6); (4) Multiplying the result of step 3 by 10 and adding 8 (i.e., 4 × 10 + 8 = 48). (5) Solving the simple division problem “48 ÷ 6” and storage of the result. (5) Combining the result of (1) and (5). Thus, like in complex multiplication, proficiency in solving simple division problems should support the solution of complex division problems.

To sum up, the most common strategy to solve simple division problems is the division by mediation strategy via retrieval of multiplication facts. Application of this strategy suggests that division and multiplication proficiency are interrelated, which has, however, not yet been examined in children after primary school. Furthermore, complex multiplication problems are usually solved by breaking them down into simpler multiplication problems. Similarly, solving complex division problems involves solving of simple division problems.

Aside from strategy use, the association between multiplication and division may also depend on skill level as suggested by the revised identical elements model (Rickard, 2005). The original identical elements model postulated that arithmetic facts with identical operands and results (i.e., identical elements) recruit a common representation in long-term memory regardless of operand order (Rickard et al., 1994; Rickard and Bourne, 1996). For instance, “3 × 4 = 12” and “4 × 3 = 12” would be stored in the same memory node, because operands differ only in their order [but see Butterworth et al. (2001), and Verguts and Fias (2005), for an alternative model explaining order effects]. However, operations with different elements would recruit independent representations. For instance, “3 × 4 = 12” and its inverse division problem “12 ÷ 3 = 4” would be stored in separate nodes, because operands and results differ. Accordingly, division problems should form a unique representation, independent from the corresponding multiplication problems. However, this assumption turned out to only be true for participants with a high skill level, as indicated in the revision of the identical elements model (Rickard, 2005). For adult participants with low and intermediate skill levels, the revised model suggests that division problems are solved by mediation via multiplication.

Whether the relationship between multiplication and division also depends on skill level in children has not been systematically investigated yet. In fact, we found only one (longitudinal) study in primary school children, which actually provided evidence contradicting this idea: De Brauwer and Fias (2009) measured arithmetic performance in multiplication and division of third graders twice a year in two consecutive school years, and of another group of children twice in second grade. To investigate the relationship between multiplication and division in primary school children, they studied the developmental parallels in performance of multiplication and division tasks as well as problem size, five and tie effects in multiplication and division. From grade 3 onward, De Brauwer and Fias (2009) found robust problem size, five, and tie effects for both operations. As these effects showed a similar time course for both operations, they suggested that their results indicate strong parallels between both operations, as to be expected when drawing on a common memory network for multiplication and division. However, contrary to the revised identical elements model, De Brauwer and Fias (2009) found that although the children's skill level increased, the strongly interconnected developmental trajectories of both operations (as measured by a similar time course of problem size, five, and tie effects) did not decrease over time. This was surprising because fourth graders in the study of De Brauwer and Fias (2009) were quite skilled in solving multiplication and division problems with error rates around 6% and reaction times below 2 s.

These converging findings might be explained by the fact that children still get faster in solving division problems after primary school (Robinson et al., 2006). Thus, the children in the study of De Brauwer and Fias (2009) might not have reached the peak of their proficiency. As a consequence, the common network for multiplication and division might only diverge in later developmental stages, namely in older children in secondary school. Moreover, developmental trajectories might be different for children with different skill levels in solving multiplication and division problems. Furthermore, the relationship between simple and complex multiplication and division problems has not yet been studied systematically.

In the present study, we aimed to investigate whether the relationship of multiplication and division after primary school depends on skill level. Skill level was indicated both by grade level (5th vs. 6th grade), school type (general or intermediate secondary school) as well as performance in arithmetic tasks. Grade level was used to investigate whether age-related differences in skill level modulate the relationship between multiplication and division. Therefore, we compared the relationship between multiplication and division in fifth grade to the relationship in sixth grade. The German educational system allowed us to divide children into groups of different skill levels depending on their secondary school type. This was possible because German children attend either one of three secondary school types based on their scholastic achievements in primary school: (1) general secondary school (“Hauptschule”), (2) intermediate secondary school (“Realschule”), or (3) grammar school (“Gymnasium”). The lowest achieving children usually attend general secondary school, the average achieving children attend intermediate secondary school, and the highest achieving children attend grammar school. Therefore, children's skill levels in multiplication and division problems should be higher in intermediate than in general secondary schools.

Overall, we expected strong developmental parallels between multiplication and division performance in line with the findings of De Brauwer and Fias (2009), that should be reflected in significant positive correlations between the two arithmetic operations. We expected a high correlation between simple multiplication and simple division, because of the usage of the division by mediation strategy by fifth and sixth graders (Robinson et al., 2006). Nevertheless, simple and complex operations should be interrelated because simple multiplications as well as divisions are sub-steps in deriving the result of the complex problems. This should be reflected by positive moderate to strong correlations. Moreover, the correlation between simple and complex division should be present even after controlling for performance in simple multiplication, because knowledge about simple division should be also relevant for solving complex division problems. Furthermore, according to the revised identical elements model (Rickard, 2005) we hypothesize lower correlations between simple multiplication and simple division performance for children with relatively higher skill levels (i.e., intermediate secondary school children, sixth graders and high performers) than children with lower skill levels (i.e., general secondary school children, fifth graders and low performers). We expect such a relationship, because for participants with low or intermediate skill levels the revised identical elements model assumes that participants rely on the division by mediation strategy. Therefore, we would expect that for participants with low or intermediate skill levels performance in simple multiplication and division should be correlated significantly. However, for participants with high skill levels the revised identical elements model assumes independent representations of multiplication and division facts. Therefore, performance of highly skilled participants in multiplication should be related less directly to performance in division resulting in relatively lower correlations than the correlation for participants with low or intermediate skill levels who are applying the division by mediation strategy.

Because of this relationship and assuming that solving complex division problems builds on solving simple division problems, we furthermore expect relatively lower correlations between simple multiplication and complex division performance for children with relatively higher skill levels than with lower skill levels. As a consequence, this pattern should also be present for correlations between simple and complex division problems. Finally, we were also interested as to whether the relationship between simple and complex multiplication depends on skill level. However, the current state of research does not allow for a directed hypothesis on this issue.

Moreover, these correlations might be influenced by other cognitive abilities. Because some children are cognitively more advanced than others, performances across many cognitive tasks are usually correlated in children. To identify the unique contribution of performance in multiplication tasks to performance in division tasks, we controlled for the influence of non-verbal intelligence and short-term memory on these interrelations. Moreover, significant correlations between multiplication and division might only suggest that arithmetic abilities are generally interrelated. Therefore, we also used children's performance in addition and subtraction tasks as a control variable to identify the specific partial (co-) variance, shared only by multiplication and division.

## Materials and methods

### Participants

We assessed the performance of 392 students in fifth and sixth grade, in two different school types of the German educational system (general secondary school, i.e., “Hauptschule,” and intermediate secondary school, i.e., “Realschule”). Students were recruited from 20 classes of nine different schools located in urban and suburban areas surrounding the city of Tuebingen (Germany) with mainly middle-class neighborhoods. We assessed the same number of classes from each school type (ten classes each). Because class sizes were smaller in general secondary schools, this resulted in a smaller sample for general than for intermediate secondary schools. In total our sample comprised 76 fifth graders (35 female, 41 male; mean age = 11.36 years, *SD* = 0.49 years) and 75 sixth graders (31 female, 44 male; mean age = 12.50 years, *SD* = 0.63 years) attending general secondary school and 112 fifth graders (56 female, 56 male; mean age = 10.88 years, *SD* = 0.41 years) and 129 sixth graders attending intermediate secondary school (100 female, 29 male; mean age = 11.89 years, *SD* = 0.45 years). Parental consent was obtained prior to the study.

### Tasks and procedure

As part of a larger project^1^, we assessed students' performance on several numerical and arithmetic tasks. Students completed simple and complex multiplication and division problems and easy and difficult addition and subtraction problems. We also assessed children's non-verbal intelligence and verbal short-term memory.

*Simple multiplication* problems involved all possible single digit multiplications excluding tie problems (e.g., 3 × 3) and multiplications including “1” or “0” as one of the operands. These exclusions were made based on research indicating that ties and problems including “1” or “0” were easier or solved with different strategies (see Campbell and Graham, 1985; Cooney et al., 1988; Siegler, 1988; Campbell and Gunter, 2002). This resulted in a set of 56 simple multiplication problems (e.g., 3 × 4). The inverse problems of these multiplications were used to create the *simple division* problems, resulting in a comparable set of 56 division problems (e.g., 3 × 4 = 12 → 12 ÷ 4 = 3).

In all *complex multiplication* problems, one of the factors was a two-digit number whereas the other one was either a single-digit or a two-digit number. Single-digit numbers ranged from 2 to 9 and two-digit numbers ranged from 12 to 19. Again, tie problems (e.g., “12 × 12”) were excluded. Results were either two- or three-digit numbers. Of the total 28 complex multiplication problems, 10 consisted of a single-digit and a two-digit factor (e.g., 7 × 16), 9 of a two-digit and a single-digit factor (e.g., 15 × 6) and 9 of 2 two-digit factors (e.g., 14 × 17).

In *complex division* problems, dividends were either two-digit or three-digit numbers. The same restrictions as for complex multiplication problems were applied in creating the division problems. Divisors and quotients were always different numbers (inversely to tie problems in multiplication). In a complex division problem, either the divisor or the quotient was a single-digit number ranging from 2 to 9, whereas the other was a two-digit number ranging from 12 to 19. Of the total 28 complex division problems, 9 consisted of a two-digit dividend and a single-digit divisor (e.g., 95 ÷ 5), 9 of a three-digit dividend and a single-digit divisor (e.g., 108 ÷ 6), 8 of a two-digit dividend and divisor (e.g., 70 ÷ 14), and 2 of a three-digit dividend and a two-digit divisor (e.g., 153 ÷ 17).

We created 36 *easy* and 36 *difficult addition* problems. Whereas none of the easy addition problems involved a carry operation (e.g., 42 + 25), half of the difficult addition problems required a carry operation (e.g., 69 + 18). *Easy* and *difficult subtraction* problems comprised 30 problems each. The easy subtraction problems did not require a borrowing operation (e.g., 54 − 31), whereas half of the difficult subtraction problems did (e.g., 63−17). Terms were single-digit, two-digit or three-digit numbers. Problems were ordered by increasing number of digits, starting with problems consisting only of single-digit numbers first and ending with problems consisting only of three-digit numbers.

Arithmetic tasks were administered to entire classrooms, as speeded paper and pencil tests with a strict time limit to prevent ceiling effects. Multiple problems were presented on each page as production tasks (e.g., “2 × 7 = __”; “21: 3 = __”) with students being instructed to solve as many problems as possible within the allotted time. Time limits were 1.5 min for simple multiplication and division tasks, 1.5 min for easy and difficult addition and subtraction tasks and 2 min for complex multiplication and division tasks. Students were not allowed to take notes. For each student, we calculated performance in simple and complex multiplication and in simple and complex division tasks as the number of correctly solved problems. Performances in both easy and difficult addition and subtraction problems were summed up to get one composite performance score for ability in solving addition and subtraction problems.

*Non-verbal intelligence* was assessed by conducting the subtest “matrices” of the Culture Fair Test (CFT-20-R, Weiss, 2006). The CFT-20-R is supposed to assess fluid intelligence, which is the capacity to think logically and solve problems in novel situations, independent of acquired knowledge. Children were instructed to solve as many of the 17 items as possible within the allotted time of 3 min.

*Verbal short-term memory* was assessed with a verbal learning and memorizing test (i.e., “Verbaler Lern- und Merkfähigkeitstest,” Helmstaedter et al., 2001). Fifteen words were consecutively read aloud to the classroom. After all words were presented, children were instructed to recall and write down as many words as possible within a time frame of 2 min.

### Analysis

Firstly, we evaluated whether performance indeed differed in the expected directions for students of different school types and of different grades. We assessed performance of children in simple and complex multiplication and division tasks. The MANOVA is the appropriate method to examine group differences when there are two or more dependent variables. Therefore, performance scores in simple and complex multiplication and division tasks were submitted to a 2 × 2 MANOVA with the independent variables school type (general secondary school vs. intermediate secondary school) and grade level (fifth vs. sixth grade) and the dependent variables performance in arithmetic tasks (simple and complex multiplication and simple and complex division).

Secondly, we computed bivariate correlations and partial correlations between performance in simple and complex multiplication and division tasks for the whole sample as well as separately for each grade level, school type and performance group. Performance groups were created by dividing our sample in low and high performance according to their skill in multiplication and division tasks using a median split^2^. Skill in multiplication and division tasks was calculated by adding children's performance scores in simple and complex multiplication and division tasks. Please note that we are fully aware of the problems of dichotomizing a continuous variable (e.g., Maccallum et al., 2002; Irwin and Mcclelland, 2003). However, dichotomizing a continuous variable can be interpreted as a more conservative test in case of bivariate analyses. Thus, if correlations are still present for low and high performers, this will indicate that the relationship between two variables is strong. Nevertheless, the median split analysis is only a supplementary analysis and findings should be interpreted with caution. In partial correlations, effects of non-verbal intelligence, verbal short-term memory and the addition and subtraction performance scores were removed. Resulting correlation coefficients were then compared applying Fisher's *r*-to-*z*-transformation in which Pearson's *r* values are converted into normally distributed *z* values. The Fisher *r*-to-*z*-transformation allows assessing the significance of the difference between two correlation coefficients found in two independent samples.

## Results

### Analyses of variance

In general, students' performance varied depending on grade level and school type. Both main effects of grade level and school type were significant [school type: Pillai-trace = 0.13, *F*_(4, 385)_ = 13.71, *p* < 0.001, η^2^_*p*_ = 0.13; grade: Pillai-trace = 0.13, *F*_(4, 385)_ = 14.12, *p* < 0.001, η^2^_*p*_ = 0.13]. The interaction between school type and grade was not significant [Pillai-trace = 0.01, *F*_(4, 385)_ = 1.40, *p* = 0.23, η^2^_*p*_ = 0.01]. Subsequent univariate analyses indicated that main effects of grade level and school type were significant in both arithmetic tasks on both difficulty levels (see Table 1; for means and SDs of control variables see Table A1; for min/max scores see Table A2). In all four tasks, sixth graders outperformed fifth graders and students from intermediate secondary schools outperformed students from general secondary schools. Thus, students' performance differed in the expected direction.

**Table 1:** **Means (SD in parenthesis) and *F*-values for general (GS) and intermediate (IS) secondary schools and fifth and sixth grade**.

	Grade				School type			
Task	5.	6.	*F*	η^2^_*p*_	*GS*	*IS*	*F*	η^2^_*p*_
Simple multiplication	14.79 (7.07)	20.45 (9.03)	46.15^**^	0.10	15.23 (8.13)	19.30 (8.56)	22.01^**^	0.05
Complex multiplication	5.44 (3.17)	7.23 (3.55)	25.91^**^	0.06	5.13 (3.26)	7.15 (3.41)	33.54^**^	0.08
Simple division	16.65 (8.21)	22.57 (10.37)	39.13^**^	0.09	15.79 (8.45)	22.20 (9.86)	44.67^**^	0.10
Complex division	2.43 (2.28)	4.05 (3.05)	28.66^**^	0.07	2.36 (2.31)	3.84 (2.97)	27.24^**^	0.07

df1 = 1, df2 = 388;

** p < 0.001.

### Correlations

When calculating correlations between all tasks for the whole sample, we found moderate to strong bivariate and partial correlations between all tasks, indicating that performance in all tasks was related (see Table 2). Moreover, after adding performance in simple multiplication as a covariate, the partial correlation between simple and complex division was still significant. It did, however, decrease from *r*_(387)_ = 0.38 to *r*_(386)_ = 0.27.

**Table 2:** **Bivariate and partial correlations (controlling for non-verbal intelligence and verbal short-term memory) between arithmetic tasks**.

	Complex multiplication	Simple division	Complex division
**BIVARIATE CORRELATIONS**			
Simple multiplication	0.59^**^	0.78^**^	0.45^**^
Complex multiplication		0.62^**^	0.56^**^
Simple division			0.53^**^
**PARTIAL CORRELATIONS**			
Simple multiplication	0.43^**^	0.70^**^	0.29^**^
Complex multiplication		0.45^**^	0.41^**^
Simple division			0.38^**^

*Bivariate correlations: df = 390, partial correlations: df = 388*,

** p < 0.001.

We then tested whether the correlation between multiplication and division performance depended on children's skill level as reflected by grade level and school type. Accordingly, we calculated partial correlations between tasks separately for each grade level, school type, and performance group and compared them using Fisher's *r*-to-*z*-transformation (see Table 3).

**Table 3:** **Partial correlations (controlling for non-verbal intelligence, verbal short-term memory and performance in solving addition and subtraction tasks) between arithmetic tasks separately for fifth and sixth grades, general (GS) and intermediate (IS) secondary schools and low-skilled and high-skilled students**.

		Grade			School type			Performance group		
Task 1	Task 2	5.	6.	*z*	*GS*	*IS*	*z*	Low	High	*z*
Simple multiplication	Simple division	0.62	0.71	−1.59	0.62	0.74	−2.04^*^	0.32	0.64	−4.12^**^
Simple multiplication	Complex division	0.16	0.27	−1.15	0.12	0.35	−2.37^**^	−0.08	0.06	−1.34
Simple multiplication	Complex multiplication	0.36	0.42	−0.69	0.39	0.44	−0.62	0.02	0.25	−2.33^**^
Simple division	Complex division	0.30	0.36	−0.71	0.20	0.44	−2.49^**^	0.14	0.16	−0.24

Partial correlations were compared using Fisher's r-to-z transformation.

GS: df = 146; IS: df = 236; 5. grade: df = 183; 6. Grade: df = 199; low-skilled: df = 191; high-skilled: df = 191;

* *p < 0.05*,

** p < 0.01(one-sided).

We observed reliably higher partial correlations between simple multiplication and simple division and between simple multiplication and complex multiplication for students from intermediate secondary schools than for students from general secondary schools. A similar pattern was present for performance groups. Partial correlations between simple multiplication and simple division were higher for higher-skilled students than lower-skilled students. However, we found no differences in partial correlation coefficients between simple multiplication and simple division for age groups.

Results for the partial correlations between simple and complex operations were different for grade level, school type and performance in multiplication and division tasks. Partial correlations between simple multiplication and complex division and between simple division and complex division differed between students from general and intermediate secondary school. As hypothesized, the partial correlation was higher for students from intermediate secondary school than from general secondary school. For performance groups, we found a significant difference for partial correlations between simple and complex multiplications with a higher correlation for high-skilled students than for low-skilled students. Other partial correlation coefficients did not differ. Moreover, we did not find any significant differences between age groups.

## Discussion

In the present study, we aimed to explore whether the strong developmental parallels between multiplication and division performance persist into secondary school and to what extent this depends on skill level. Therefore, we assessed the performance of fifth and sixth graders of two secondary school types (general vs. intermediate secondary school) of the German educational system in multiplication and division problems. In line with our hypotheses, we found that sixth graders outperformed fifth graders and students from intermediate secondary schools outperformed students from general secondary schools. Thus, skill levels differed both between age groups (fifth vs. sixth graders) as well as between school types (general vs. intermediate secondary school). In the following section, we will first discuss age-related differences before elaborating on school type differences and finally, we will discuss the specificity of correlations between multiplication and division tasks.

### Age-related influences

Our results indicated that strong developmental parallels between multiplication and division persist into fifth and sixth grade of secondary school. In particular, simple multiplication and division were strongly interrelated, even after controlling for non-verbal intelligence, verbal short-term memory and arithmetic performance in addition and subtraction tasks. Thus, we suggest that, comparable to the study of Robinson et al. (2006) students mostly relied on the division by mediation strategy when solving division problems. According to Robinson et al. (2006), fifth and sixth graders applied this strategy in about 50% of all problems, making it the most applied strategy in these age groups. Extending the results of De Brauwer and Fias (2009), who found that simple multiplication and division were interrelated until grade 4, we found evidence that they are still interrelated reliably in grade 6.

Furthermore, simple and complex tasks were interrelated reliably, suggesting that knowledge about simple problems is indeed recruited for solving complex problems. More specifically, we found that students' knowledge about simple division contributed to their performance in solving complex division problems even after controlling for performance in simple multiplication.

However, bivariate and partial correlations were lower for complex tasks, which might be caused by children's overall lower performance in complex tasks. Thus, lower correlations might be due to floor effects reducing variability. Nevertheless, performance in complex tasks varied within a sufficiently large range (i.e., 0–13 correctly solved problems). This speaks against a general floor effect for complex problems. However, it would be interesting to evaluate the current results by giving children more time for the complex tasks allowing them to solve more problems.

Comparable to the study of De Brauwer and Fias (2009), our findings cannot be accounted for by the revised identical elements model (Rickard, 2005). According to the model, only students at low and intermediate skill levels should apply the division by mediation strategy. Contrary to this hypothesis, all students were quite skilled at solving multiplication and division problems as indicated by their fast solution times. For multiplication problems, fifth graders took about 6.26 s per problem and sixth graders took about 4.50 s per problem. For division problems, fifth graders took about 5.35 s per problem and sixth graders took about 4.13 s per problem^3^. They were somewhat slower than students in the studies of Robinson et al. (2006) and De Brauwer and Fias (2009), who needed less than 3 s to solve multiplication and division problems. However, in the study by Robinson et al. (2006), students had to answer verbally and in the study of De Brauwer and Fias (2009), verification and number-matching tasks were used. In our study, students had to write down answers, which obviously took more time than responding verbally or pressing a button. Interestingly, students in our study were faster at solving simple division than simple multiplication problems, which seems to contradict our implication that they relied on an indirect strategy. However, again this finding can be explained by the nature of the tasks we used. Most simple multiplication problems required students to write down two digits, whereas all simple division problems required them to write down only one digit. Thus, slightly faster solution times for simple division problems might be due to less time needed to write down the solutions in simple division compared to simple multiplication problems.

Importantly, we did not find significant differences in correlation coefficients between fifth and sixth graders. Thus, we did not find age-related differences in skill level to modulate the relationship between multiplication and division. We assume that high correlations between simple multiplication and division tasks indicated that students relied on the division by mediation strategy. Thus, the reliance on the division by mediation strategy cannot explain age-related performance differences, because the observed correlations seem to indicate that in both age groups children relied on the strategy to a similar extent.

### School type differences

Performance differences between students from general and intermediate secondary schools allowed us to look at how the relationship between multiplication and division depends on differences in skill level between secondary school types. We found that students from intermediate secondary schools outperformed students from general secondary schools. In line with the revised identical elements theory (Rickard, 2005), we hypothesized that correlations between simple multiplication and division tasks should be lower for intermediate secondary school children than for general secondary school children. However, we found the exact opposite. Partial correlations were even higher for students from intermediate secondary schools, suggesting that their multiplication and division memory network was even more closely related. This interpretation was corroborated by similar findings for the partial correlation between simple multiplication and complex division. Solving complex division problems involves splitting them up into simple division problems. If students who are better at solving multiplication problems apply the mediation by division strategy, they should also be better at solving complex division problems. Thus, our results suggest that students from intermediate secondary schools who performed better in simple and complex division relied more frequently or used conceptually or procedurally more consistently the inverse relationship between multiplication and division.

The relationship between skill level and simple and complex problems was less clear. For the correlation between simple multiplication and complex multiplication we found that skill level as indexed by differences between school types had no influence. Nevertheless, the significant correlations between simple and complex multiplication indicate that students from general as well as from intermediate secondary schools rely on their knowledge about simple multiplications when solving complex multiplications. Thus, our finding suggests that students from different school types rely on their knowledge about simple multiplication to a similar amount. However, students from intermediate schools did not seem to rely more heavily on their knowledge about simple multiplications.

In contrast to multiplication, we found that skill level as indexed by differences between school types moderated the correlations between simple and complex division. Thus, it seems that students with higher skill levels (i.e., students from intermediate secondary schools) relied more heavily on their skills in simple division to solve complex division problems. However, we found a similar pattern between simple multiplication and simple division and between simple multiplication and complex division. Therefore, one possible interpretation is that students from intermediate secondary schools made greater use of the division by mediation strategy in both simple and complex division problems by retrieval of simple multiplication facts.

### Performance dependent differences

We also examined whether correlations between multiplication and division tasks depended on students' performance in multiplication and division tasks. Therefore, we allocated students to one of two performance groups.

In accordance with findings for different school types, we found higher correlations for more skilled students. Higher correlations suggest that more skilled students seemed to rely more heavily on the inverse relationship between simple multiplication and division, and thus, they used the division by median strategy more often. Thus, we found confirmatory evidence that skill level modulates the relationship between simple multiplication and division in secondary school children.

For the relationship between simple and complex tasks, findings for performance groups differed from findings for school types. Correlations between simple multiplication and complex division and between simple division and complex division were not modulated by skill level. Thus, more skilled students did not seem to rely more heavily on their knowledge about simple multiplication and simple division when solving complex division problems. Moreover, correlation coefficients between simple multiplication and complex division were close to zero suggesting that students do not rely on their knowledge about simple multiplication when solving complex division problems. However, there was a reliable partial correlation between simple multiplication and complex division before allocating students to different groups [*r*_(386)_ = 0.29, *p* < 0.001]. Thus, the disappearance of this association may be explained by the method used to create two performance groups. It has been shown repeatedly that a median split can reduce correlation coefficients between two variables as it dichotomizes a continuous variable and thus reduces variability which is detrimental in correlation analyses (Maccallum et al., 2002; Irwin and Mcclelland, 2003). In the present case, this might have led to the reduced correlation between simple multiplication and complex division.

Furthermore, we observed that skill level modulated the relationship between simple and complex multiplication. Correlation coefficients were higher for more skilled students suggesting that they relied more on their knowledge about simple multiplications when solving complex multiplications. Moreover, a near to zero correlation coefficient for the low performance group seems to imply that students from this group did not rely on their knowledge about simple multiplication when solving complex multiplication problems. Because knowledge about simple multiplication is necessary for solving complex multiplications, this finding is quite implausible and might again stem from creating dichotomous performance groups from a continuous variable by means of a median split.

Taken together, the performance group analysis confirmed our finding of skill related differences as indexed by differences between school types for the relationship between simple multiplication and division. Moreover, contrary to the revised identical elements model (Rickard, 2005) we did not find any evidence that the relationship between simple and complex multiplication and division tasks was weaker for high performers than low performers.

### Specificity of correlations

In this study, we used general cognitive and arithmetic abilities as control variables to identify the unique covariance between performance in multiplication and division tasks. All partial correlations remained significant after controlling for intelligence, short-term memory and general arithmetic ability (as measured by performance in addition and subtraction tasks). However, they were lower than the bivariate correlations suggesting that general cognitive and arithmetic capabilities contribute to bivariate correlations between multiplication and division. Thus, bivariate correlations between these operations may overestimate the unique covariance of both operations, because better cognitive and arithmetic capabilities may lead to better performances in both operations.

The partial correlations allowed us to identify which tasks are most closely interrelated. After controlling for general cognitive and arithmetic abilities, the correlation between simple multiplication and simple division was higher than all other correlations. Complying with the findings of Robinson et al. (2006), one possible interpretation of this finding is that fifth and sixth graders mostly rely on their knowledge about multiplication facts to solve simple division problems. In contrast, we found lower correlations between simple multiplication and complex multiplication and division tasks after controlling for general cognitive and arithmetic abilities. Hence, general cognitive and arithmetic abilities as well as procedural knowledge might be more important than knowledge about multiplication facts when solving complex tasks.

However, a possible limitation of our study is that we only inferred strategies used from correlations we observed between multiplication and division performance. While our results provide conclusive evidence that children seemed to make use of the division by mediation strategy, a more direct investigation of their strategy use would be beneficial. One way to do so might be the consideration of verbal reports as used by Robinson et al. (2006). Moreover, correlations do not allow for causal conclusions. Therefore, even if it seems implausible from a developmental point of view (division is introduced after multiplication), children might have relied on their knowledge about simple division when solving simple multiplication problems.

## Conclusions

In the current study we were interested (i) whether the close developmental parallels between multiplication and division persist beyond primary school, (ii) whether similar developmental parallels can be observed for simple and complex problems, (iii) whether these relationships are influenced by skill level, and (iv) whether the correlations are specific and not driven by general cognitive or arithmetic abilities. By collecting data of fifth and sixth graders attending general or intermediate secondary schools we operationalized skill level both within as well as between age groups. In general, our findings provide converging evidence for the importance of the inverse relationship between multiplication and division. In line with this interpretation, moderate to strong partial correlations between multiplication and division performance - even after controlling for short-term memory, intelligence and other arithmetic abilities, indicate that students seem to recast division problems as multiplication problems. Importantly, skill level as indexed by school type influenced this relationship, as students from intermediate secondary schools seem to draw more heavily on multiplication fact retrieval in division as indicated by higher correlations between the two operations. This finding was confirmed for the relationship between simple multiplication and division when analyzing performance dependent difference. Yet, we did not find any evidence that age-related differences in skill level modulated the relationship between multiplication and division. These findings are hard to reconcile with the predictions made by the identical elements model, which claims that mediation of division by multiplication should decrease as skill level increases.

### Conflict of interest statement

The authors declare that the research was conducted in the absence of any commercial or financial relationships that could be construed as a potential conflict of interest.

## Footnotes

^1^In addition to the pen and paper tasks on arithmetic, we also collected data about students' proficiency in orthography. Furthermore, students also completed computerized numerical tasks which addressed research questions different than the ones presented here.

^2^We wish to thank Reviewer Elena Ise for suggesting this analysis.

^3^Time spent per problem was calculated by dividing the number of solved problems by the total allotted time.

## Appendix

**Table A1:** **Means (SD in parenthesis) of control variables for general (GS) and intermediate (IS) secondary schools and fifth and sixth grade**.

	School type		Grade	
Task	*GS*	*IS*	5.	6.
Verbal short-term memory	6.99 (2.35)	8.07 (1.77)	7.40 (1.95)	7.89 (2.17)
Non-verbal intelligence	8.26 (2.63)	8.92 (2.73)	8.60 (2.60)	8.72 (2.81)
Score in addition and subtraction	45.79 (9.94)	51.29 (8.73)	47.64 (9.22)	50.58 (9.72)

**Table A2:** **Minimum (min), maximum (max), mean and SD of each variable**.

	Min	Max	Mean	*SD*
Simple multiplication	1	51	17.73	8.62
Complex multiplication	0	17	6.37	3.49
Simple division	0	55	19.73	9.84
Complex division	0	13	3.27	2.82
Verbal short-term memory	0	14	7.65	2.08
Non-verbal intelligence	0	14	8.67	2.71
Score in addition and subtraction	16	75	49.17	9.58
